# Health Equity Assessment Toolkit (HEAT): software for exploring and comparing health inequalities in countries

**DOI:** 10.1186/s12874-016-0229-9

**Published:** 2016-10-19

**Authors:** Ahmad Reza Hosseinpoor, Devaki Nambiar, Anne Schlotheuber, Daniel Reidpath, Zev Ross

**Affiliations:** 1 World Health Organization, 20 Avenue Appia, 1211 Geneva 27, Switzerland; 2 Public Health Foundation of India, 47, Sector 44, Gurgaon, 122002 India; 3 Jeffrey Cheah School of Medicine and Health Sciences, Monash University, Jalan Lagoon Selatan, Bandar Sunway, 46150 Selangor, DE Malaysia; 4 ZevRoss Spatial Analysis, 120 N Aurora St, Suite 3A, Ithaca, NY 14850 USA

## Abstract

**Background:**

It is widely recognised that the pursuit of sustainable development cannot be accomplished without addressing inequality, or observed differences between subgroups of a population. Monitoring health inequalities allows for the identification of health topics where major group differences exist, dimensions of inequality that must be prioritised to effect improvements in multiple health domains, and also population subgroups that are multiply disadvantaged. While availability of data to monitor health inequalities is gradually improving, there is a commensurate need to increase, within countries, the technical capacity for analysis of these data and interpretation of results for decision-making. Prior efforts to build capacity have yielded demand for a toolkit with the computational ability to display disaggregated data and summary measures of inequality in an interactive and customisable fashion that would facilitate interpretation and reporting of health inequality in a given country.

**Methods:**

To answer this demand, the Health Equity Assessment Toolkit (HEAT), was developed between 2014 and 2016. The software, which contains the World Health Organization’s Health Equity Monitor database, allows the assessment of inequalities within a country using over 30 reproductive, maternal, newborn and child health indicators and five dimensions of inequality (economic status, education, place of residence, subnational region and child’s sex, where applicable).

**Results/Conclusion:**

HEAT was beta-tested in 2015 as part of ongoing capacity building workshops on health inequality monitoring. This is the first and only application of its kind; further developments are proposed to introduce an upload data feature, translate it into different languages and increase interactivity of the software. This article will present the main features and functionalities of HEAT and discuss its relevance and use for health inequality monitoring.

## Background

The 2015 launch of the Sustainable Development Goals (SDGs) marks a paradigm shift in the global discourse on poverty and development. It is widely recognised that the pursuit of sustainable development cannot be accomplished without addressing inequality. Goals 5 (gender equality) and 10 (reducing inequality within and among countries) reflect this shift [1–3], aptly conveyed in the Secretary General’s simple appeal to “leave no-one behind” [1, 4]. A key step towards achieving the goal of leaving no-one behind is ensuring the availability of data for all countries, including the least developed, on key development indicators disaggregated by dimensions of inequality like income, sex, age, and geographic location [5, 6]. The disaggregated data can then be used to track and benchmark progress within and between countries. More specifically, Goal 3 calls for ensuring healthy lives and promoting well-being for all at all ages and universal health coverage, making explicit a commitment to health equity [2, 5, 7–10]. Monitoring health inequalities can identify progress over time, highlighting the impact of health policies, programs, and interventions on the most-disadvantaged subgroups. It can also serve as a warning system when health differences between population subgroups widen [11, 12]. Health inequality monitoring entails collecting, analysing, interpreting, and reporting health disaggregated data. While data availability is gradually improving, there is a commensurate need to increase, within countries, the technical capacity for analysis of these data and interpretation of results for decision-making [13].

It has been found that routine reviews of health sector performance (such as annual health sector reviews) tend to report national averages and occasionally averages for sub-populations (e.g. urban and rural residents). This level of data disaggregation does not allow for a more critical analysis of inequality, including trends or benchmarking, that could assist with the policy/programme design or refinement [14]. Indeed, in the absence of appropriately disaggregated data, there is a danger that national health averages could improve without any improvement in health inequality [11, 15].

The World Health Organization (WHO) has been working closely with national governments to build capacity for health inequality monitoring [13]. Training workshops on measuring and monitoring health inequalities have covered a large number of countries, giving an opportunity for government decision-makers to interpret data on health inequalities and to carry out priority-setting based on this appraisal. Throughout this process, it has been recognised that interpretation and priority-setting is greatly enhanced if analysts are equipped with a user-friendly tool that can be used to synthesize and visualize disaggregated data as well as summary measures of inequality (like differences and ratios [11]). It is also helpful if analysts can see the latest data on the status of health inequality and see change over time (i.e. whether inequality has been increasing or decreasing as distinct from what average trends are showing).

In the initial training workshops, the pivot function in Microsoft Excel was used to show disaggregated data in tables and charts. Summary measures were calculated using the publicly available Health Disparity Calculator software (HD*Calc) (seer.cancer.gov/hdcalc) [16, 17]. While this software combination could be used to perform the necessary analyses, it was inflexible. Excel had only limited interactivity features and did not allow for quick comparisons of multiple dimensions of inequality. HD*Calc required the creation/importation of specific file formats for analysis, and could only provide results for one indicator and one dimension at a time while importing data from an Excel file. This precluded the comparison of one indicator against another or the comparison of the same indicator with multiple inequality dimensions. These elements of interactivity were identified as crucial for the interpretation of the data and subsequent priority-setting. Over multiple workshops it became clear that there was a demand for a toolkit with the computational ability to display disaggregated data and summary measures in an interactive and customisable fashion that would facilitate interpretation and reporting of health inequality in a given country. To answer this demand, the Health Equity Assessment Toolkit (HEAT) was developed. During its development, HEAT was tested in capacity-building workshops on health inequality monitoring in the WHO Eastern Mediterranean Region (February 2015) and the WHO Region of the Americas (December 2015). In these workshops, there was strong endorsement of and appreciation for the software from participants representing health ministries and statistical agencies as well as trainers from reputed academic institutions. Feedback was received on the interface, technical aspects, aspects related to training and facilitation of the use of the software, as well as confirmation of functionalities to be included in HEAT going forward.

HEAT is intended to be used primarily by those who are familiar with health information systems and have basic skills in interpreting health-related data. This may include technical staff (for example, in ministries of health and statistical offices), public health professionals, policy-makers, researchers, and students. This article will present the main features and functionalities of HEAT and discuss its relevance and use for health inequality monitoring.

## Implementation

HEAT was developed using the free and open source statistical software R (https://www.r-project.org) and the R package shiny (https://cran.r-project.org/web/packages/shiny). R is a free and open source software environment for statistical computing and graphics that operates in a Windows, Mac OS X, and Linux environment. Key R packages used in the tool implementation include dplyr for data analysis and management as well as the packages ggplot2, RColorBrewer, grid and gridExtra for graphing of the multi-dimensional data [18–22]. Shiny is a free, open source, extensible web applications framework for R that allows the creation of a rich, interactive web interface for querying and summarising data as tables, free text, or graphs. A shiny application can either operate on a local machine using a standard web browser to manage the interaction with a local instance of R, or it can operate on an internet connected server (http://www.shinyapps.io). The HEAT source code was published under the GNU General Public License Version 2 (https://www.gnu.org/licenses/gpl-2.0) and is freely available through GitHub (https://github.com/WHOequity/HEAT-1.0). Summary features of HEAT are provided in Table 1.

**Table 1:** Features of HEAT software

Feature	Description
General	
Software	HEAT was developed using the R statistical software and the R package shiny. Additional R packages used to support HEAT include: ggplot2, dplyr, RColorBrewer, grid and grid Extra.
License	GNU GPL version 2.
Availability	HEAT is available as an online application and as a standalone version for use offline.
Compatibility	The online version can be accessed using any web browser on all desktop or laptop computers and mobile devices (minimum screen size of 7.9” is recommended).
	The standalone version can be accessed on all computers with a Windows or Macintosh operating system.
Installation	The online version requires no installation.
	The standalone version is available in a zip folder and needs to be extracted and saved to the computer’s hard drive. The extracted HEAT folder contains portable versions of the R statistical software and the web browser Mozilla Firefox, which are required to run HEAT, but do not require any installation. The standalone version can simply be launched by double-clicking the start file.
Built-in database	
Disaggregated data	HEAT contains the WHO Health Equity Monitor database. Its 2015 update includes estimates and 95 % confidence intervals for more than 30 reproductive, maternal, newborn and child health indicators, disaggregated by five dimensions of inequality (economic status, education, place of residence, subnational region and child’s sex (where applicable)) from Demographic and Health Surveys and Multiple Indicator Cluster Surveys conducted in 94 countries between 1993 and 2013. The database is updated regularly.
Summary measures of inequality	HEAT calculates 15 summary measures of inequality and their 95 % confidence intervals based on analytic and/or bootstrap methods.
Export options	
Tables	Data can be exported as text files with values separated by comma or by tab. Users can choose their preferred field separator.
Graphs	Data can be visualised in bar graphs, line graphs and scatterplots. Users can adjust the height and width of graphs, specify axes ranges and add titles and axis labels. In addition, users can display 95 % confidence intervals. Graphs can be exported as pdf, jpg or png files.
Supporting material	
User manual	The user manual provides detailed information on how to set up and work with HEAT. Each feature of the toolkit is explained in detail and recommendations are made on how best to assess and interpret the data.
Technical notes	The technical notes provide detailed information about the data displayed in HEAT, including the disaggregated data from the WHO Health Equity Monitor database and the 15 summary measures of inequality that were calculated based on the disaggregated data.
Indicator compendium	The indicator compendium includes a comprehensive definition of each indicator included in the WHO Health Equity Monitor database.

HEAT is available as an online application and as a standalone version for use offline. Both versions can be accessed through the WHO website (http://www.who.int/gho/health_equity/assessment_toolkit/). The online version can be accessed using any web browser on all desktop or laptop computers and mobile devices (a minimum screen size of 7.9 inches is recommended). The standalone version can be used on computers with Windows or Macintosh operating systems (separate packages are available for Windows and Macintosh). The standalone packages can be downloaded as .zip files that include portable versions of R and Mozilla Firefox, and do not require any additional software or installation.

HEAT comes pre-installed with the WHO Health Equity Monitor database [23]. The 2015 update of the database draws on Demographic and Health Survey (DHS) as well as Multiple Indicator Cluster Survey (MICS) data from 94 countries, mostly low- or middle-income, collected between 1993 and 2013. For almost three quarters of the countries, data are available for at least two time points. The database includes more than 30 Reproductive, Maternal, Newborn and Child Health (RMNCH) indicators covering both health interventions and health outcomes. Data have been disaggregated by five dimensions of inequality: economic status, education, place of residence, subnational region and child’s sex (where applicable). The database is updated regularly. In addition, fifteen widely used summary measures of inequality have been calculated in HEAT - seven absolute measures and eight relative measures relevant for health inequality monitoring [11, 14]. An overview of all summary measures, including their definitions, formulas and application to the dimensions of inequality is provided in Table 2.

**Table 2:** Overview of summary measures and dimensions

			Dimension of inequality				
Summary measure	Definition	Formula^a^	Economic status	Education	Place of residence	Sex	Subnational region
Absolute measures							
Absolute concentration index (ACI)	The ACI is a complex, weighted measure of inequality that indicates the extent to which a health indicator is concentrated among the disadvantaged or advantaged, on an absolute scale.	$ACI={\displaystyle \sum_j}{p}_j\left(2{X}_j-1\right){y}_j$	✓	✓			
Between-group variance (BGV)	The BGV is a complex, weighted measure of inequality that shows the squared difference between each subgroup and the national level, on average. The BGV is sensitive to large deviations from the national level (by use of squaring).	$BGV={\displaystyle \sum_j}{p}_j{\left({y}_j-\mu \right)}^2$					✓
Difference (D)	The D is a simple measure of inequality that shows the absolute inequality between two subgroups.	*D* = *y*_*high*_ − *y*_*low*_	✓	✓	✓	✓	✓
Mean difference from best performing subgroup (MDB)	The MDB is a complex, weighted measure of inequality that shows the difference between each subgroup and the best performing subgroup, on average.	$MDB={\displaystyle \sum_j}{p}_j\left|{y}_j-{y}_{ref}\right|$					✓
Mean difference from mean (MDM)	The MDM is a complex, weighted measure of inequality that shows the absolute difference between each subgroup and the national level, on average.	$MDM={\displaystyle \sum_j}{p}_j\left|{y}_j-\mu \right|$					✓
Population attributable risk (PAR)	The PAR is a complex, weighted measure of inequality that shows the potential for improvement in the national level of a health indicator that could be achieved if all subgroups had the same level of health as a reference subgroup.	*PAR* = *y*_*ref*_ − *μ*	✓	✓	✓	✓	✓
Slope index of inequality (SII)	The SII is a complex, weighted measure of inequality that represents the absolute difference in predicted values of a health indicator between the most-advantaged and most-disadvantaged (or vice versa for adverse health outcome indicators), while taking into consideration all the other subgroups – using an appropriate regression model.	*SII* = *v*_1_ − *v*_0_ for favourable health intervention indicators; *SII* = *v*_0_ − *v*_1_ for adverse health outcome indicators	✓	✓			
Relative measures							
Index of disparity (IDIS)	The IDIS is a complex measure of inequality that shows the proportional difference between each subgroup and the national level, on average.	$IDIS=\frac{1}{n}*\frac{{\displaystyle {\sum}_j}\left|{y}_j-\mu \right|}{\mu }*100$					✓
Kunst-Mackenbach index (KMI)	The KMI is a complex, weighted measure of inequality that represents the ratio of predicted values of a health indicator of the most-advantaged to the most-disadvantaged (or vice versa for adverse health outcome indicators), while taking into consideration all the other subgroups – using an appropriate regression model.	*KMI* = *v*_1_/*v*_0_ for favourable health intervention indicators; *KMI* = *v*_0_/*v*_1_ for adverse health outcome indicators	✓	✓			
Mean log deviation (MLD)	The MLD is a complex measure of inequality that takes into account the population share of each subgroup. The MLD is sensitive to large deviations from the national level (by use of logarithm).	$\mathrm{M}\mathrm{L}\mathrm{D}={\displaystyle \sum_j}{p}_j\left(- \ln \left(\frac{y_j}{\mu}\right)\right)*1000$					✓
Population attributable fraction (PAF)	The PAF is a complex, weighted measure of inequality that shows the potential for improvement in the national level of a health indicator, in relative terms, that could be achieved if all subgroups had the same level of health as a reference subgroup.	$PAF=\frac{PAR}{\mu }*100$	✓	✓	✓	✓	✓
Ratio (R)	The R is a simple measure of inequality that shows the relative inequality between two subgroups.	*R* = *y*_*high*_/*y*_*low*_	✓	✓	✓	✓	✓
Relative concentration index (RCI)	The RCI is a complex, weighted measure of inequality that indicates the extent to which a health indicator is concentrated among the disadvantaged or the advantaged, on a relative scale.	$RCI=\frac{ACI}{\mu }*100$	✓	✓			
Relative index of inequality (RII)	The RII is a complex, weighted measure of inequality that represents the relative difference (proportional to the national level) in predicted values of health indicator between the most-advantaged and most-disadvantaged, while taking into consideration all the other subgroups – using an appropriate regression model.	$RII=\frac{SII}{\mu }$	✓	✓			
Theil index (TI)	The TI is a complex measure of inequality, that takes into account the population share of each subgroup. The TI is sensitive to large deviations from the national level (by use of logarithm).	$TI={\displaystyle \sum_j}{p}_j\frac{y_j}{\mu } \ln \frac{y_j}{\mu }*1000$					✓

*y*_*j*_ is the estimate for subgroup j, *y*_*high*_ is the estimate for the highest subgroup, *y*_*low*_ is the estimate for the lowest subgroup, *y*_*ref*_ is the estimate for the reference subgroup, *p*_*j*_ is the population share for subgroup j, ${\boldsymbol{X}}_{\boldsymbol{j}}={\displaystyle \sum_{\boldsymbol{j}}}{\boldsymbol{p}}_{\boldsymbol{j}}-0.5{\boldsymbol{p}}_{\boldsymbol{j}}$ relative rank of subgroup j, *μ* is the national average, *v*_0_ is the predicted value of the hypothetical person at the bottom of the social-group distribution (rank 0), *v*_1_ is the predicted value of the hypothetical person at the top of the social-group distribution (rank 1), *n* is the number of subgroups

^a^For detailed information about these formulas, please refer to the HEAT software’s technical notes, available at: http://www.who.int/gho/health_equity/heat_technical_notes.pdf

The software displays disaggregated data and summary measures of inequality in tabular and graphical format, allowing for interactivity (e.g. multiple indicators or inequality dimensions) may be viewed at the same time, views can alternate between latest status and a chosen number of years for a single country.

## Results & discussion

The features of this software were conceptualized and developed between 2014 and 2016 in conjunction with capacity-building activities on health inequality monitoring involving multiple workshops with participants from a large number of countries. Since HEAT automates the computational tasks of calculating summary measures of inequality and visually depicts disaggregated data and summary measures of inequality for the user, the advantage is that data is ready for interpretation, allowing users to focus on the assessment of inequalities to move from data to action.

**Fig. 1:**
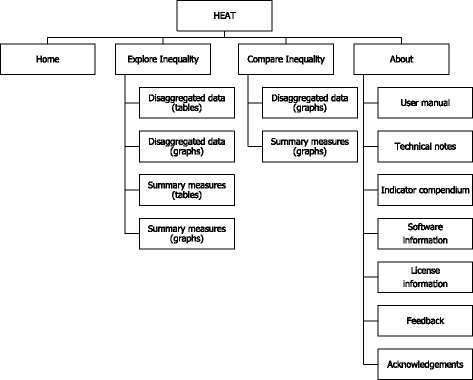
Structure of toolkit

To enable this, the HEAT interface has four main tabs: Home (which is the starting/homepage view), Explore Inequality, Compare Inequality, and About. The homepage provides an introduction and citation information. The About page includes tabs displaying the User manual, Technical notes, Indicator compendium, Software information, License information, Feedback information and Acknowledgements. The structure of the tookit is indicated in Fig. 1; more details on the remaining two key tabs, Explore Inequality and Compare Inequality, are provided below.

### Explore inequality

Explore Inequality comprises four tabs, displayed in a horizontal panel at the top, to view the data in tabular and graphic format: Disaggregated data (tables), Disaggregated data (graphs), Summary measures (tables), and Summary measures (graphs). Disaggregated data (tables) presents a table with data on chosen health indicators by population subgroups (classified by dimension of inequality) in a selected country of interest for a given year, or multiple years. Disaggregated data (graphs) presents horizontal line graphs (equiplots [24]) or bar graphs with health data for population subgroups for one or more survey years in a selected country of interest. Summary measures (tables) presents a table with chosen summary measures of inequality for a selected country of interest for a given year, or multiple years. Summary measures (graphs) presents line graphs or bar graphs for a chosen summary measure of inequality for one or more survey years in a selected country of interest.

**Fig. 2:**
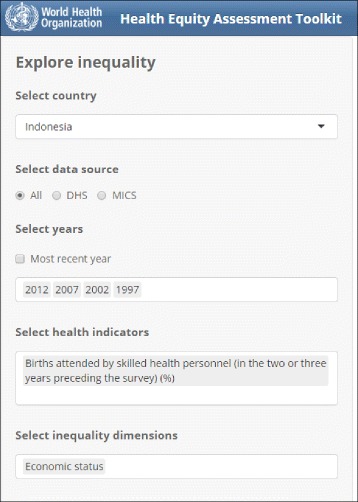
Screenshot of side panel

In all tabs, there is a panel on the left that allows users to toggle and interact with the views in the screen, including choosing the country, data source(s), year(s), health indicator(s) and inequality dimension(s). In Summary measures tabs, the summary measure(s) may additionally be chosen. Multiple selections are allowed, where relevant, and default selections afford the user a view that can then be toggled and manipulated using the options along the top and left (the User Manual has recommendations on what optimal views are from the standpoint of interpretation). A screenshot of the left panel is depicted in Fig. 2. Each page allows downloading of data in a variety of commonly used formats (.csv and .tsv for tables and .pdf, .png, and .jpg for graphs).

When either the Disaggregated data (tables) or Summary measures (tables) tabs is selected, there are ‘Table Options’ on the left panel that may be manipulated to define/change table content. Users may add or remove variables, such as the 95 % confidence interval (lower and upper bounds). The number of decimal places visible may also be altered. For the Summary measures (tables) view, it is also possible to alter the multipliers applied to estimates, as appropriate for interpretation.

**Fig. 3:**
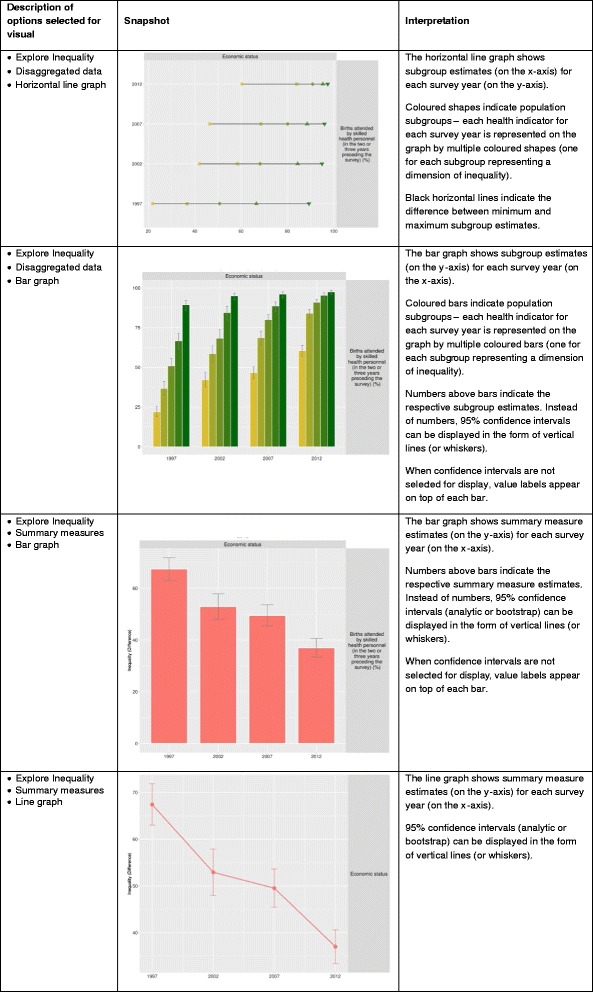
Guide to graphs under the Explore Inequality tab

When either the Disaggregated data (graphs) or Summary measures (graphs) tabs is selected, there are ‘Graph Options’ on the left panel that may be manipulated to define/change graph content. This includes changing the type of graph (users can choose between a bar graph or line graph/equiplot), graph height and width, axis range, as well as graph and axis titles. For bar graphs, users can additionally alternate between displaying the estimate value on top of bars or displaying the 95 % confidence intervals (in the form of whiskers). For line graphs in the Summary measures (graphs) tab, it is also possible to include whiskers for 95 % confidence intervals. Screenshots of graph views available under this tab, along with their interpretation are depicted in Fig. 3.

### Compare inequality

In Compare Inequality, users can compare the situation of their chosen country with that of comparators using disaggregated data or summary measure graphs. Following a similar logic to Explore Inequality, the panel on the left allows one to toggle and interact with the views in the screen, including choosing the country, data source(s), year, health indicator, and the inequality dimension the user wants to view. In Summary measures (graphs), the summary measure may also be chosen. In addition to this, there are benchmarking options allowing users to choose comparator countries by World Bank income group and WHO region, which creates a shortlist of countries that may further be added/removed from the graph. There is also an option to modify the range of years (0 to 5 years) that will be considered in the comparison. For example, if a range of 0 years is selected for a country of interest for the year 2007, only those countries that also have data from 2007 will be presented. If a range of 5 years is selected for the same country and year, any country that has data for the indicator in question between 2002 and 2012 will be included. Graph options similar to other graph tabs are provided to change graph height, width, axis range, as well as graph and axis titles.

As with Explore Inequality, default selections afford the user a view that can then be toggled and manipulated using the options along the top and left (the User Manual has recommendations on what optimal views are from the standpoint of interpretation). Each page allows downloading of data in a variety of commonly used formats (.csv and .tsv for tables and .pdf, .png, .jpg for graphs).

**Fig. 4:**
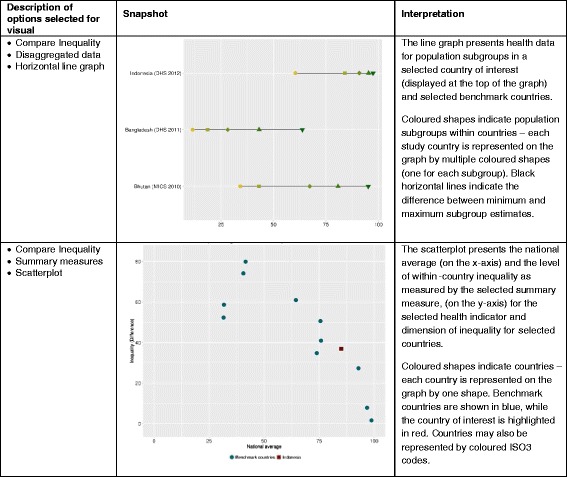
Guide to graphs under the Compare Inequality tab

Screenshots of graph views available under this tab, along with their interpretation are depicted in Fig. 4.

### Case study

In April 2016, HEAT was used in a capacity building workshop on health inequality monitoring in Indonesia consisting of 30 participants from the Ministry of Health, the National Statistical Office, academia, and other UN agencies.

Using HEAT, participants first explored the current and past state of inequality in RMNCH in Indonesia using the Explore Inequality tab. By visualising disaggregated data and summary measures in tables and graphs, participants could identify different patterns and levels of inequality for different indicators and dimensions. For example, it was found that economic-related inequality in coverage of births attended by skilled health personnel greatly decreased between 1997 and 2012, but large absolute differences in coverage between the richest and poorest population subgroups remained in 2012. On the contrary, existing gaps in demand for family planning satisfied were closed completely in the same period. Participants also observed that for certain indicators, the situation varied between different dimensions of inequality. As an example, while economic-related inequality in measles immunization coverage remained unchanged over time, differences between urban and rural residents were eliminated.

Participants then went on to compare the situation in Indonesia with that of other middle-income countries from the South-East Asia and Western Pacific Regions using the Compare Inequality tab. Looking at births attended by skilled health personnel, for example, there were countries that have achieved almost complete coverage, such as the Maldives and Mongolia, while other countries reported even larger inequalities than Indonesia. Again, the situation varied between different health indicators and dimensions of inequality.

HEAT facilitated an initial assessment of inequalities in RMNCH in Indonesia and showed where inequalities existed [25]. In this sense, it can be considered as a priority-setting tool to identify the largest within-country health inequalities. This being said, HEAT is a toolkit to assist health inequality monitoring and serves as a warning system. It does not depict multivariate analyses of inequality or explain why inequalities exist, for which further in-depth quantitative and qualitative studies are required [11].

### Future developments

Going forward, an upload data feature will be incorporated into the software so that instead of the Health Equity Monitor database, data meeting pre-defined specifications may be uploaded for health inequality analyses. It is also proposed to add additional interactive features to this software, like pop-up features to display or expand information shown in a data point or to rank and annotate data based on interpretation. There are several R packages that enable the creation of interactive graphics using html widgets or other approaches; these include Plotly, Highcharter, rCharts and others. Interactive maps using the Leaflet or Highcharter packages may also be considered. In addition, there are R packages that provide bindings to the Google Translate API that may allow the translation of the tool in to other languages.

## Conclusion

HEAT comes at a critical point of renewal and re-invigoration in global health cooperation and national priority-setting in health. In the post-2015 era, the importance of disaggregated data for sustainable development is acknowledged [5, 6], and this is one major attempt using software that facilitates exploring and comparing health inequality data in a user-friendly, interactive, and fully-flexible format. There is no similar application in the market of which we are aware.

## Abbreviations

DHS: Demographic and Health Survey
HEAT: Health Equity Assessment Toolkit
MICS: Multiple Indicator Cluster Survey
RMNCH: Reproductive, Maternal, Newborn and Child Health
SDGs: Sustainable Development Goals
WHO: World Health Organisation
